# The Medicago SymCEP7 hormone increases nodule number via shoots without compromising lateral root number

**DOI:** 10.1093/plphys/kiad012

**Published:** 2023-01-19

**Authors:** Ariel Ivanovici, Carole Laffont, Estíbaliz Larrainzar, Neha Patel, Courtney S Winning, Han-Chung Lee, Nijat Imin, Florian Frugier, Michael A Djordjevic

**Affiliations:** Division of Plant Sciences, Research School of Biology, The Australian National University, Canberra, Australian Capital Territory 2601, Australia; University of Paris-Saclay, CNRS, INRAE, University Paris-Cité, Univ. d’Evry, Gif-sur-Yvette, France; Sciences Department, Institute for Multidisciplinary Research in Applied Biology (IMAB), Universidad Pública de Navarra, Pamplona 31006, Spain; Division of Plant Sciences, Research School of Biology, The Australian National University, Canberra, Australian Capital Territory 2601, Australia; Division of Plant Sciences, Research School of Biology, The Australian National University, Canberra, Australian Capital Territory 2601, Australia; Division of Plant Sciences, Research School of Biology, The Australian National University, Canberra, Australian Capital Territory 2601, Australia; Division of Plant Sciences, Research School of Biology, The Australian National University, Canberra, Australian Capital Territory 2601, Australia; School of Science, Western Sydney University, Penrith, New South Wales 2751, Australia; School of Biological Sciences, Faculty of Science, The University of Auckland, Auckland, New Zealand; University of Paris-Saclay, CNRS, INRAE, University Paris-Cité, Univ. d’Evry, Gif-sur-Yvette, France; Division of Plant Sciences, Research School of Biology, The Australian National University, Canberra, Australian Capital Territory 2601, Australia

## Abstract

Legumes acquire soil nutrients through nitrogen-fixing root nodules and lateral roots. To balance the costs and benefits of nodulation, legumes negatively control root nodule number by autoregulatory and hormonal pathways. How legumes simultaneously coordinate root nodule and lateral root development to procure nutrients remains poorly understood. In Medicago (*Medicago truncatula*), a subset of mature C-TERMINALLY ENCODED PEPTIDE (CEP) hormones can systemically promote nodule number, but all CEP hormones tested to date negatively regulate lateral root number. Here we showed that Medicago *CEP7* produces a mature peptide, SymCEP7, that promotes nodulation from the shoot without compromising lateral root number. Rhizobial inoculation induced *CEP7* in the susceptible root nodulation zone in a Nod factor-dependent manner, and, in contrast to other *CEP* genes, its transcription level was elevated in the ethylene signaling mutant *sickle*. Using mass spectrometry, fluorescence microscopy and expression analysis, we demonstrated that SymCEP7 activity requires the COMPACT ROOT ARCHITECTURE 2 receptor and activates the shoot-to-root systemic effector, miR2111. Shoot-applied SymCEP7 rapidly promoted nodule number in the pM to nM range at concentrations up to five orders of magnitude lower than effects mediated by root-applied SymCEP7. Shoot-applied SymCEP7 also promoted nodule number in White Clover (*Trifolium repens*) and Lotus (*Lotus japonicus*), which suggests that this biological function may be evolutionarily conserved. We propose that SymCEP7 acts in the Medicago shoot to counter balance the autoregulation pathways induced rapidly by rhizobia to enable nodulation without compromising lateral root growth, thus promoting the acquisition of nutrients other than nitrogen to support their growth.

## Teaser

The Medicago SymCEP7 peptide hormone has a symbiosis-associated activity that increases nodule number when applied to legume shoots in the picomolar to nanomolar range.

## Introduction

In the past ∼100 million years, legumes evolved an ability to form nitrogen (N)-fixing root nodules through a symbiotic relationship with soil bacteria, termed “rhizobia” (Sprent, 2008). Root nodules provide legumes with a reliable N source under limiting conditions. Since the energetic cost of supporting N-fixing rhizobia is high, legumes carefully control nodule number primarily through autoregulatory- and ethylene-dependent processes to balance the carbon cost needed to sustain rhizobia with the benefits of N fixation (Ferguson et al., 2019). Over past decades it has become increasingly clear that legumes have coopted and rewired preexisting genetic pathways to enable the evolution of nodule development (Gonzalez-Rizzo et al., 2006; Schiessl et al., 2019; Soyano et al., 2019) and to control nodule number (Djordjevic et al., 2015; Imin et al., 2018; Ferguson et al., 2019; Gautrat et al., 2020). How legumes simultaneously co-ordinate nodule and lateral root number and development to secure all the nutrients required to support their growth remains poorly understood.

Nod Factors (NFs) secreted by *Sinorhizobium spp*. trigger nodule development in Medicago (*Medicago truncatula*) by binding to plasma membrane localized NF receptors in growing root hair cells. In plate-grown Medicago, susceptibility to nodule formation is restricted primarily to a circa 15 mm nodulation zone defined by the position of the root tip at the time of inoculation and, hence, most successful root hair infections and nodule initiations occur during a 48-hour period (Mohd-Radzman et al., 2016). Therefore, all processes that promote and then progressively restrict nodule number occur in this tight nodulation zone and temporal window (Djordjevic et al., 2015; Mohd-Radzman et al., 2016). Therefore, focusing on the nodulation zone enables the assessment of the activity of hormones that either promote or inhibit the competency of roots for nodulation and which determine the final nodule number.

Apart from the well-known genes that are required locally in roots for nodule formation, such as the pivotal Symbiosis (Sym) pathway transcription factor *NODULE INCEPTION* (*NIN*) (Liu et al., 2019), legumes also control root nodule number and root hair infections via systemic pathways. First, root-to-shoot mobile peptide hormones, encoded by specific members of the *C-TERMINALLY ENCODED PEPTIDE* (*CEP*) and *CLAVATA3-LIKE* (*CLE*) gene families, act systemically to control both root nodule number and rhizobial infections positively and negatively, respectively (Mortier et al., 2010; Imin et al., 2013; De Bang et al., 2017; Imin et al., 2018; Tsikou et al., 2018; Gautrat et al., 2019, 2020; Laffont et al., 2020; Zhu et al., 2020). *CEP* genes induced under low-N conditions promote susceptibility to rhizobial infection and nodulation and their corresponding mature CEP hormones interact with the leucine-rich repeat receptor-like kinase, COMPACT ROOT ARCHITECTURE 2 (CRA2) (Imin et al., 2013; Gautrat et al., 2020). Conversely, a subset of *CLE* genes, which are up-regulated by rhizobia and/or high nitrate, suppress rhizobial infections and nodule development through autoregulation of nodulation mechanisms and their corresponding mature CLE hormones interact with the SUPER NUMERIC NODULES (SUNN) receptor (Mortier et al., 2010; Saur et al., 2011; Imin et al., 2018; Mens et al., 2021; Moreau et al., 2021; Okuma and Kawaguchi, 2021).

Ethylene-dependent pathways mediated through SICKLE (SKL), the Medicago orthologue of Arabidopsis ETHYLENE INSENSITIVE2 (EIN2), also negatively control the number of rhizobial infections (Penmetsa et al., 2008). Therefore, *skl* mutants are hyper-infected and form a higher nodule number, and the expression of early Sym pathway genes is also elevated in this mutant (Larrainzar et al., 2015). Mohd-Radzman et al. (2016) and Zhu et al. (2020) showed that the Medicago CEP1 hormone negatively controls SKL-dependent signaling through the CRA2 receptor. Therefore, the combined interaction of CEP, CLE, and ethylene-mediated pathways positively and negatively balance the regulation of Sym pathway gene expression, respectively, to fine tune the overall nodule number that form in the nodulation zone (Mohd-Radzman et al., 2016; Laffont et al., 2019; Gautrat et al., 2020; Laffont et al., 2020).

The Medicago *CEP* multigene family gives rise to various mature 15-amino acid hormones (Imin et al., 2013; Ogilvie et al., 2014; Mohd-Radzman et al., 2015; Patel et al., 2018). The primary amino acid sequence, as well as the position and number of hydroxyl proline post-translational modifications of mature 15 amino acid CEP hormones, affect their biological activity. For example, non-hydroxylated CEP hormones, or incorrectly processed CEP hormones possessing additional *N*-terminal amino acid extensions, have weaker or no biological activity on Medicago (Mohd-Radzman et al., 2015; Patel et al., 2018). Previous in vivo studies identified the mature 15 amino acid CEP hormones derived the Medicago *CEP1, CEP2, CEP5*, and *CEP8* genes using axenic hairy root cultures (Mohd-Radzman et al., 2015; Patel et al., 2018). The biologically active Medicago CEP hormones identified to date in vivo have two classes of activity. All 15 amino acid CEP hormones identified and tested inhibit lateral root number, but most of these have no effect on nodule number (e.g. CEP2 and CEP5 species), whereas a few species not only decrease lateral root number but also increase nodule number (e.g. CEP1 species) (Patel et al., 2018; Zhu et al., 2021). Such inhibition of lateral root number and foraging by CEP could be considered counterproductive for Medicago roots to secure soil nutrients other than N.

The current understanding of CEP signaling in Medicago centers on studies with the CEP1 hormone. The application of the CEP1 hormone to roots attenuates ethylene signaling and thus increases the number of infection threads, infection pockets, and nodules (Mohd-Radzman et al., 2015; Mohd-Radzman et al., 2016; Zhu et al., 2020). Lee et al. (2021) recently showed that the binding of CEP1 to shoot vascular cells requires the CRA2 receptor. Currently, because *CEP* genes are predominantly expressed in roots, CEP peptides are directly exposed to roots to test their activity where they have peak nodule number promoting activity in the µM concentration range. The requirement for such a high concentration, however, is somewhat paradoxical since prior quantitative experiments using mass spectrometry demonstrated that the concentration of CEP hormones in vivo is in the low or sub nM range (Mohd-Radzman et al., 2015). Therefore, CEP hormone concentrations in the µM range could be physiologically irrelevant. In addition, Gautrat et al. (2020) proposed that the CEP1 hormone, which accumulates under low N, binds to CRA2 in the shoot to promote competence for nodulation by inducing *premiR2111d* expression, which increases the level of the mature miR2111 shoot-to-root systemic effect or that subsequently negatively regulates *TOO MUCH LOVE* (*TML*) transcription in the root.

Recent evidence suggests that *CEP7* has a symbiosis-associated expression pattern during the early stages of infection of the Medicago root by rhizobia (Jardinaud et al., 2016; Laffont et al., 2020). Indeed, rhizobia rapidly induce *CEP7* expression, and the NIN transcription factor binds to its promoter. We also recently showed that a synthetic CEP7 hormone with proline hydroxylation at positions 4 and 11 increased nodule number and decreased lateral root number when applied to roots at µM levels (Laffont et al., 2020); however the presence of CEP7 with this pattern of proline hydroxylation has not been verified using in vivo studies. In this study, we identified Medicago *CEP7* as a major player for promoting nodulation in the nodulation zone. We used mass spectrometry to define the predominant hormone structure for CEP7 in vivo, and showed it had a unique biological activity compared to all other Medicago CEPs previously studied since it promoted nodule number without negatively impacting lateral root number. Due to its symbiosis-associated activity, we named this peptide hormone variant SymCEP7. We tested if the putative root-to-shoot translocation of SymCEP7 could be bypassed by directly applying it to shoots at physiologically-relevant concentrations in the pM to nM range (Mohd-Radzman et al., 2015) and determined if the binding of SymCEP7 to shoot vascular tissue required CRA2. We undertook a kinetic analysis to determine how quickly shoot-applied SymCEP7 influences root nodule number compared to root-applied SymCEP7. These data indicate that SymCEP7 acts through the CRA2 receptor in shoots to regulate nodulation systemically. Finally, we explored the evolutionary conservation of Medicago SymCEP7 using phylogenetic analyses and by applying nM range doses to the shoots of the determinate-nodulating legume Lotus (*Lotus japonicus*) and the indeterminate-nodulating legume, White Clover (*Trifolium repens*). This analysis revealed that shoot-applied SymCEP7 also has a nodule number promoting activity in these species. Overall, these data suggest a model where the nodulation-promoting activity of SymCEP7 during Medicago infection counterbalances the inhibiting effects of the nodulation autoregulation pathway without inhibiting lateral root number.

## Results

### *CEP7* is induced rapidly in the nodulation zone and is hyper-induced in *skl*

Prior RNA-seq data were used to monitor the expression of *CEP* encoding genes over a 48-hour period post-rhizobial inoculation in the wild-type (A17), *nfp* (defective in NF perception) and *skl* mutant roots (Larrainzar et al., 2015). *CEP7* expression depended on rhizobial inoculation and the NF receptor, NFP. *CEP7* expression elevated 4-fold in the wild type between 12 and 24 hours post inoculation (hpi) before it attenuated at later time points, whereas it increased up to 8-fold in *skl* roots at 48 hpi without showing attenuation (Supplemental Figure 1). None of the other *CEP* genes tested showed rhizobial or NF-dependent expression and their transcriptional levels were not elevated in *skl* over the 48-hour time series (Supplemental Figure 1).

**Figure 1:**
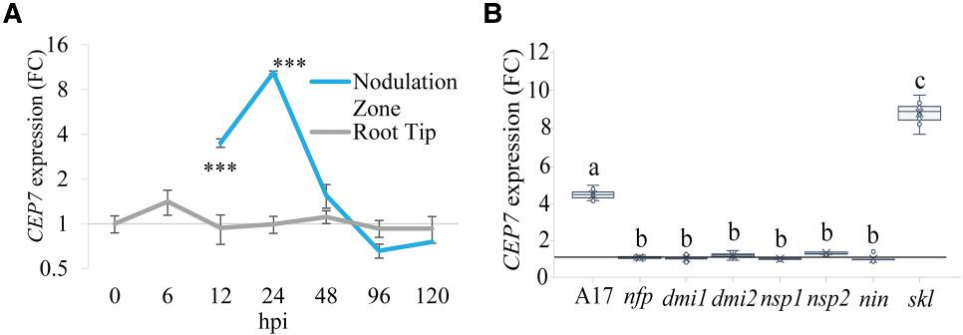
*CEP7* expression is up-regulated in the nodulation zone during the early stages of infection and is hyper-expressed in *skl*. A, RT-qPCR analysis of *CEP7* expression fold change (FC) in wild-type plants grown on N-free Fåhraeus medium for five days and then inoculated with Sm1022. RNA was extracted from root tips or from the nodulation zone tissues as defined in Mohd-Radzman et al. (2016), at seven time points following inoculation. The interaction between root regions over time was analyzed using Linear Mixed Effects modelling and significant differences are shown with asterisks, error bars show standard deviation (SD) (***P < 0.001, *n* = 4, biological replicates each contain 27–36 roots). B, RT-qPCR analysis of *MtCEP7* expression in wild type (A17), Sym pathway defective mutants, and *skl* at 24 hpi. Gene expression is shown relative to the 0 hpi time point for each mutant, respectively using the *ubi* gene (Medtr_4g091580) as a control. Data are plotted as open circles, centre lines show the median; box limits indicate the 25th and 75th percentiles, crosses represent samples means, whisker boundaries show the 1.5 interquartile range. Significant differences are shown with letters (Two-way ANOVA, *P* < 0.001, *n* = 6, biological replicates contain 12–24 roots, three experimental repeats each with six biological repeats). Hpi, hours post inoculation.

To identify the region of the root where *CEP7* was induced, we measured relative expression levels in either the nodulation zone of the root or root tips (Figure 1A). Reverse transcription quantitative PCR (RT-qPCR) analyses confirmed that *CEP7* expression follows a sharp transient induction pattern between 12 and 24 hpi in the nodulation zone, before attenuating at subsequent time points (Figure 1A). The duration of root *CEP7* expression (Figure 1A) was consistent with the transient peak of *CEP7* expression observed in wild type plants using RNA-seq. RT-qPCR analysis additionally demonstrated that there was no induction of *CEP7* expression in the non-nodulating mutants, *nfp, dmi1, dmi2, nsp1, nsp2*, or *nin* mutants, at 24 hpi (Figure 1B). By contrast, the increase of *CEP7* transcripts in the *skl* mutant (Figure 1B) was consistent with its upregulation during the early stages of symbiosis and its elevated expression in *skl* observed in the RNA-seq (Supplemental Figure 1). To further support the NF-dependent response observed, we used RT-qPCR to show that *CEP7* was induced by NFs isolated from sinorhizobia (*S. meliloti*) but not from the non-compatible symbiont *Bradyrhizobium japonicum.* The non-nodulating sinorhizobia strain SL44 (deleted for *nodDABC* genes) also failed to induce *CEP7* (Supplemental Figure 2A).

### *CEP7* produces a domain-1 peptide hormone variant (SymCEP7) with a symbiosis-associated biological activity

**Figure 2:**
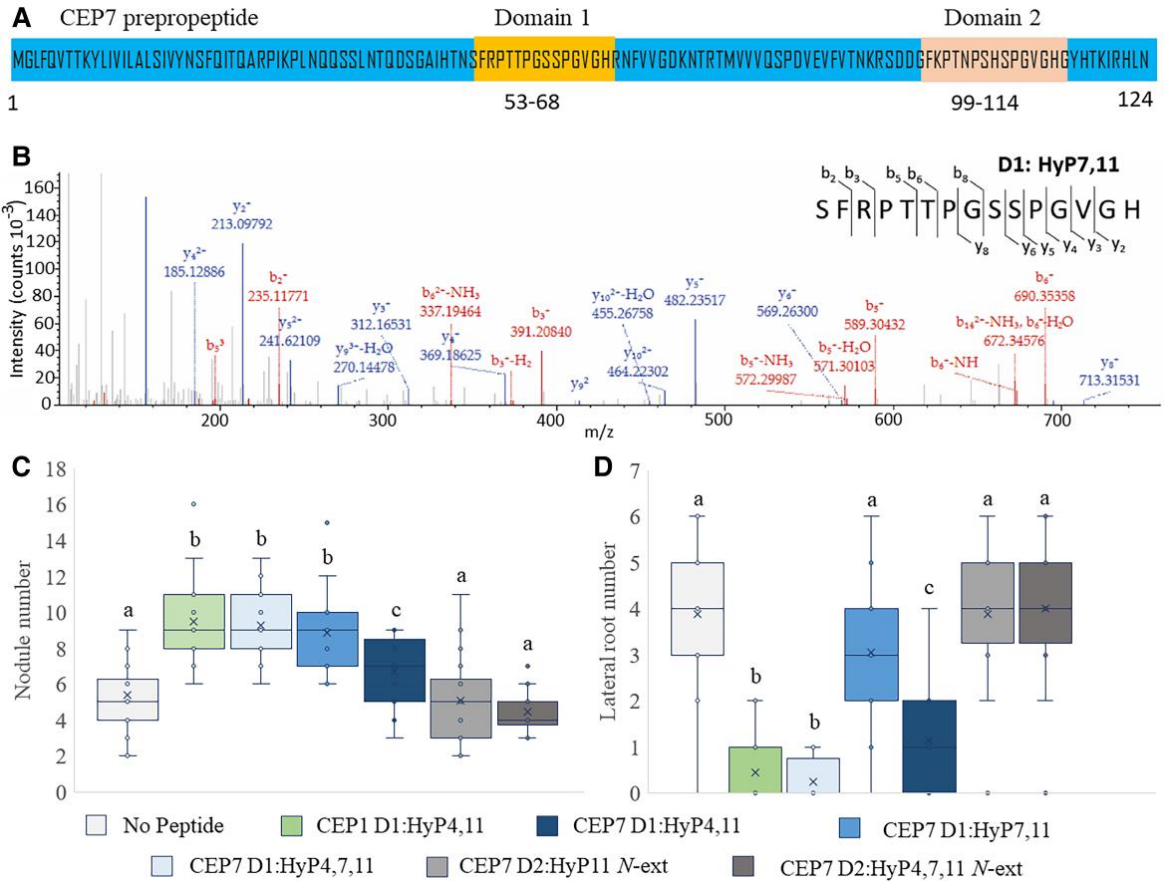
*CEP7* produces a symbiosis-associated hormone structure that promotes nodule number without affecting lateral root number. A, Predicted *CEP7* gene product showing the two CEP domains (positions 53-68 and 99-114). B, High accuracy MS-MS spectrum of the predominant *CEP7*-derived hormone species identified in vivo in hairy root culture exudates (Patel et al., 2018). The spectrum shows the ion intensity values for the matched *y* and *b* ions corresponding to the domain 1 CEP7 peptide hormone. The spectrum is consistent with proline hydroxylation at position 7 and 11 of the identified 15 amino acid product. C, D, Plants were grown on N-free medium and inoculated with Sm1022. Peptides were added to the agar medium just prior to pouring plates so that they contacted the roots only. Nodules (C) and lateral roots (D) were scored 14 days post inoculation. In (C) and (D), data are plotted as open circles, centre lines show the median; box limits indicate the 25th and 75th percentiles, crosses represent samples means, whisker boundaries show the 1.5 interquartile range. Significant differences are shown with letters (Two-way ANOVA, *P* < 0.001, *n* = 18–24 plants, two experimental repeats each with 18–24 biological repeats).

The structures of peptide hormones produced in vivo from native *CEP7* transcription were determined using mass spectrometry (MS) (Mohd-Radzman et al., 2015; Patel et al., 2018). The most strongly ionized CEP7 peptide (ion intensity ∼1.4 × 10^5^) corresponded to the 15 amino acid CEP domain 1 (amino acid residues 53–68 in Figure 2A)(Delay et al., 2013; Ogilvie et al., 2014) with proline hydroxylation at position 7 and 11 (i.e. CEP7 D1:HyP7, 11 Figure 2B). The other putative CEP7 domain 1 peptide, which had proline hydroxylations at positions 4, 7, and 11, was detected at a 5 to 6-fold lower ionization intensity (∼ 0.25 × 10^5^), whereas the other two species identified corresponded to the CEP domain 2 of CEP7 with four additional amino acids located to the N-terminal side of the 15 amino acid domain (Supplemental Figure 3A–C). Based on the greater ion intensity of CEP7 D1:HyP7, 11 relative to the other species detected, it is likely to be one of the most abundant CEP7 domain 1 species.

To test the biological activity of these structural variants, they were synthesized and applied to roots (Figure 2B and Supplemental Figure 3 A–C). The biological activity of these peptides was assessed relative to CEP7D1:HyP4, 11 and CEP1D1: Hyp 4, 11, which were used as positive controls as they were tested in similar functional assays previously (Figure 2, C and D; Imin et al., 2013; Mohd-Radzman et al., 2015, 2016; Patel et al., 2018; Laffont et al., 2020). Strikingly, CEP7D1:Hyp7, 11 had a symbiosis-associated activity when compared to all other characterized Medicago CEPs: it increased nodule number, but unlike all other Medicago CEP hormones tested previously, it did not reduce lateral root number (Figure 2C and D). Since this result indicates that CEP7 D1:HyP7,11 has a symbiosis-associated biological activity, we refer to it as SymCEP7. A comparison of the biological activity of SymCEP7 relative to CEP7D1:HyP4, 11 and CEP1D1HyP4, 11 highlighted its symbiosis-associated activity. Consistent with previous reports, CEP7D1:HyP4, 11 and CEP1D1HyP4, 11 both increased nodule number and inhibited lateral root number when applied to roots (Figure 2C and D) (Imin et al., 2013; Laffont et al., 2020). Of the three other CEP7 hormones detected in vivo, CEP7D1: HyP4, 7, 11 raised nodule number and reduced lateral root number, whereas the other CEP7 domain 2 structures were biologically inactive (Figure 2, C and D) most likely due to the four amino acid *N*-terminal extensions, which we had shown previously to diminish the activity of CEP hormones relative to the corresponding 15 amino acid species (Patel et al., 2018). As expected, the application of SymCEP7 to the roots of *nin*, *dmi2*, or *cra2* failed to increase nodule number (Supplemental Figure 2B), whereas in the wild type, SymCEP7 application to the roots increased the number of nodules (Figure 2C; Supplemental Figure 2B) as well as infection threads and infection pockets (Supplemental Figure 4A–C).

### A fluorescent version of SymCEP7 (FITC-SymCEP7) is biologically active and its association with the shoot vasculature requires the CRA2 receptor

**Figure 3:**
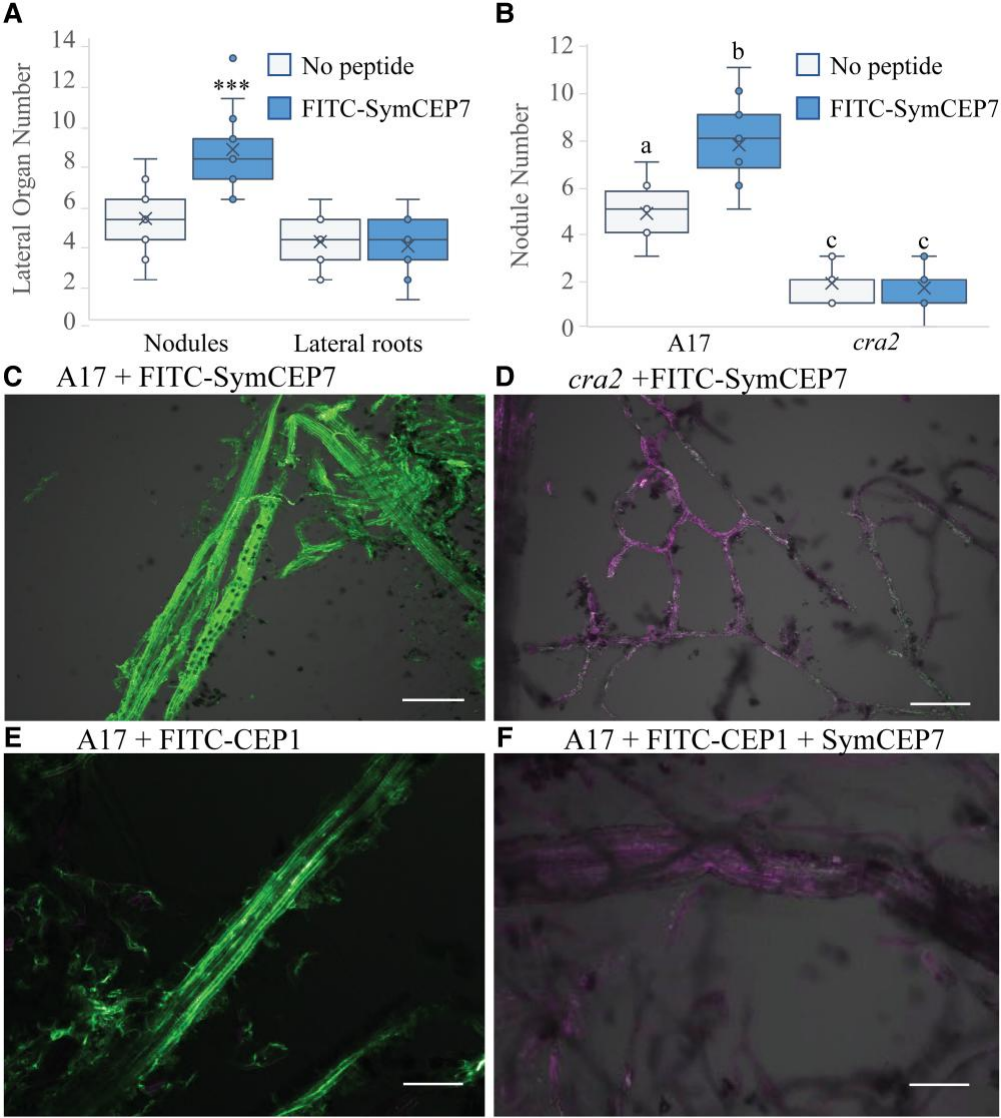
FITC-SymCEP7 increases nodule number and its activity depends on CRA2. A, FITC-SymCEP7 was added to wild type (A17) roots throughout their growth period. Plants were inoculated with Sm1022 and nodules and lateral roots were scored 14 days post inoculation. B, A17 and *cra2* mutant plants were grown with or without SymCEP7 and inoculated with Sm1022. As in panel (A), peptides were added to the agar medium just prior to pouring plates so that they contacted the roots throughout the experiment. Nodules were scored 14 dpi. Significant differences are shown with asterisks (Student's T-test, ***P < 0.001, *n* = 15-18 roots, two experimental repeats). In (A) and (B) data are plotted as open circles, centre lines show the median; box limits indicate the 25th and 75th percentiles, crosses represent samples means, whisker boundaries show the 1.5 interquartile range. Significant differences are shown with letters (Two-way ANOVA, *P* < 0.001, *n* = 14–18 roots, two experimental repeats). (C, D) Medicago leaf vascular tissue was isolated from A17 (C) and *cra2* (D), incubated with FITC-SymCEP7, washed, cross-linked, and observed using confocal microscopy. Vasculature tissues were incubated with FITC-SymCEP7 for 30 minutes and washed to remove non-specific binding before formaldehyde cross-linking. FITC fluoresces at a distinct excitation wavelength from the auto–fluorescence due to cell wall related phenolic compounds. E, F, Medicago leaf vascular tissue was isolated from A17 and incubated with (E) FITC-CEP1 D1:Hyp4,11 (1 µM) only, or (F) FITC-CEP1 D1:Hyp4,11 (0.5 µM) and SymCEP7 (0.5 µM) in a peptide competition assay (added for 30 minutes) before washing and cross-linking. The FITC fluorescence maximum is at 525 nm and auto–fluorescence due to cell wall related phenolic compounds peaks at ~600 nm, scale bar = 100 µm.

Prior experiments showed that the CEP-dependent increase in nodule number depends on the activity of the CRA2 receptor in the shoot, and that *CRA2* is expressed in the vasculature of both shoots and roots (Huault et al., 2014; Laffont et al., 2020). To confirm that SymCEP7 interacts with CRA2 in the shoot, we first synthesized FITC-SymCEP7 and showed that it increased root nodule number in a CRA2-dependent manner when added to roots, with no effect on lateral roots, as expected (Figure 3A and B). We then determined if FITC-SymCEP7 associates with shoot vascular tissue in a CRA2-dependent manner using the in vivo binding method developed by Lee et al. (2021). FITC-SymCEP7 bound to the Medicago shoot vasculature of wild type leaves but not to the shoot vasculature derived from *cra2* (Figure 3C and D). We then conducted a peptide competition assay, which showed that SymCEP7 displaced the binding of FITC-CEP1D1: HyP4, 11 from the vascular tissue (Figure 3E and F). Since FITC-CEP1D1: HyP4, 11 binding to the wild type shoot vasculature depends on CRA2 (Lee et al., 2021), this suggested that both peptides compete with each other for the same binding site and that CRA2 is a receptor for both peptides (Lee et al., 2021).

### Shoot-applied SymCEP7 increases nodule number in roots without affecting lateral root number, and induces the expression of *miR2111*

**Figure 4:**
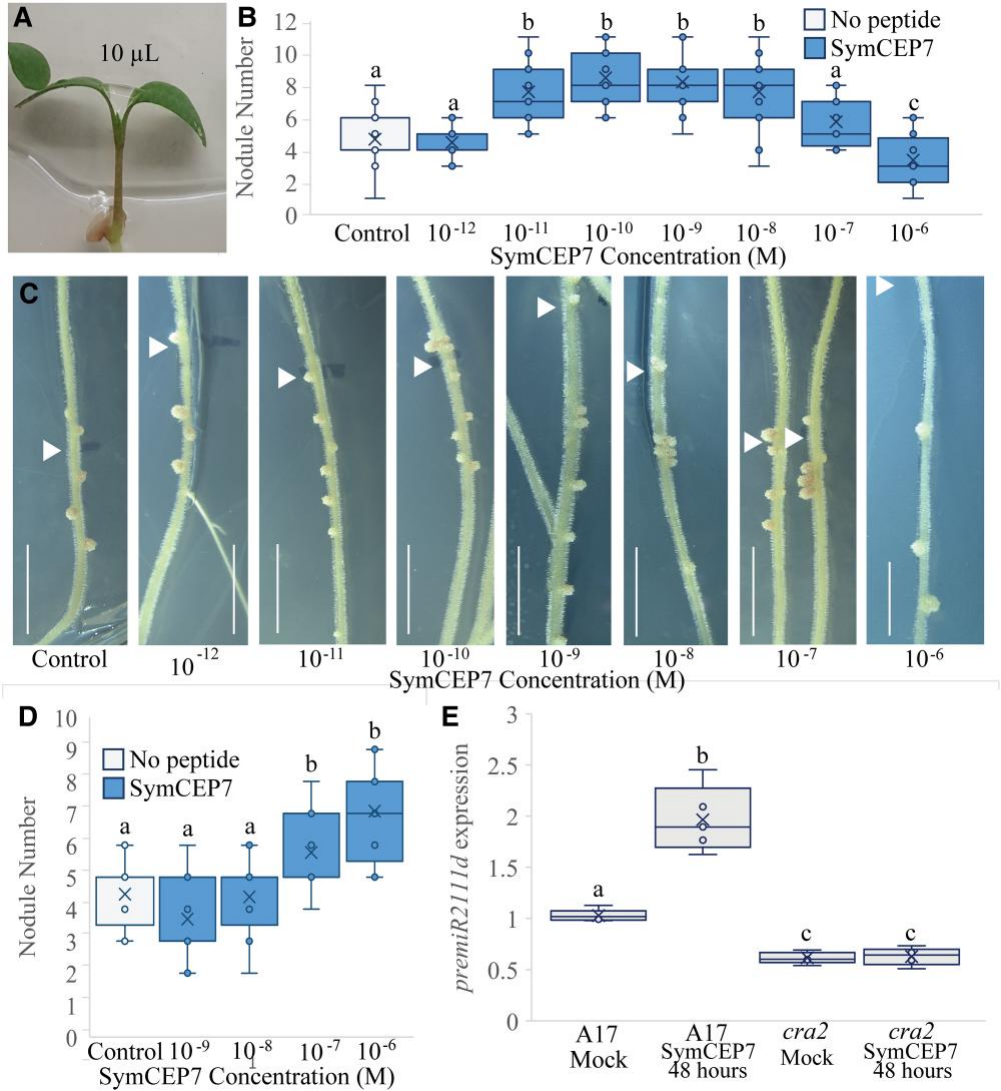
Shoot-applied SymCEP7 increases nodule number in a concentration and CRA2-dependent manner and induces *premiR2111d* expression. A–C, A 10 µl drop of SymCEP7 peptide solution was applied to the petioles of the cotyledons of four-day old wild-type (A17) seedlings (A) and rhizobia were inoculated onto the roots at the same time (B, C). The volume of SymCEP7 was replenished to 10 µl daily for five days. Roots were inoculated with Sm1022 (OD_600_ = 0.01) four days after germination. Nodule number was scored 14 days post inoculation. Two-way ANOVA was used to compare treatments (*P* < 0.001, *n* ≥ 24 plants, three experimental repeats each with 20–24 biological repeats). C, shows representative images of (B) (scale bars = 1 cm, arrow denotes position of the root tip at the time of inoculation). D, SymCEP7 was incorporated into the agar medium and subsequently four-day old seedlings were inoculated with Sm1022 (OD_600_ = 0.01). (Two-way ANOVA, *P* = 0.013, *n* = 18 plants). E, RT-qPCR analysis of *premiR2111d* expression in wild type and *cra2.* Plants were grown on N-free Fåhraeus plates for four days prior to the addition of 10 µl of 10 nM SymCEP7 or water to the shoots (as in A). Wild type and *cra2* shoots were harvested after 48 hours. Significant differences are shown with letters (Two-way ANOVA, *n* = 5, biological replicates each consist of 8–15 shoots, three experimental repeats each with five biological repeats). In (B, D), and (E), data are plotted as open circles, centre lines show the median; box limits indicate the 25th and 75th percentiles, crosses represent samples means, whisker boundaries show the 1.5 interquartile range.

All prior experiments used root-applied CEP hormones but because SymCEP7 associated with the shoot vascular tissue of wild type but not of *cra2* mutants, we tested if a direct application to the shoot could control root nodule number. This was done by applying 10 µl droplets of SymCEP7 to the petioles of the cotyledons of four-day old plate-grown seedlings when the first leaf was emerging (Figure 4A). We applied SymCEP7 at concentrations ranging from 10^−12^ M to 10^−6^ M, and rhizobia were applied to the roots at the same time. Strikingly, shoot-applied SymCEP7 promoted nodule number at concentrations up to five orders of magnitude lower than root-applied SymCEP7. Specifically, SymCEP7 increased root nodule number most effectively when applied in the mid-pM to -nM range (10^−11^ M to 10^−8^ M) relative to the no peptide control (Figure 4B and C). By contrast, at 10^−7^ M, shoot-applied SymCEP7 imparted no significant difference in nodule number relative to the no peptide control, and it inhibited and delayed nodulation at 10^−6^ M (Figure 4B and C). These results reflected a typical hormonal dose-response of shoot-applied SymCEP7 activity on nodulation. In contrast to shoot applications, root-applied SymCEP7 required µM levels to induce a maximum increase of the root nodule number, and its nodule number promoting activity was not significant at 10^−8^ M or lower concentrations (Figure 4D). Finally, we showed that the positive effect on nodule number imparted by shoot-applied SymCEP7 at nM concentrations was abolished in *cra2* (Supplemental Figure 5B). Collectively, these data reveal that shoot-applied SymCEP7 mediates an increase in nodule number at concentrations in the mid pM to nM range, and that this activity depends on the shoot localized CRA2 receptor.

Since CEP1 hormones similarly act through CRA2 in the shoot, we also tested CEP1 D1:HyP4,11 applications (at 10^−8^ M) to the shoot. This peptide increased nodule number without having a significant effect on lateral root number (Supplemental Figure 5A). This provided an independent approach to that published previously (Imin et al., 2013; Huault et al., 2014; Laffont et al., 2019), to show that CEP1 inhibits lateral root number locally in the root but promotes nodule number from the shoot at lower concentrations than when applied to the root.

Since the low-N induced CEP1 hormones promote nodulation through the activation of the miR2111 shoot-to-root systemic effector when applied to roots in the µM range (Laffont et al., 2019), we tested if applying SymCEP7 to the shoot at 10^−8^ M affected the expression of *premiR2111d* in the shoot. The expression of *premiR2111d* was demonstrated previously to be representative of the expression pattern of the different miR2111 precursors family (Gautrat et al., 2020). A 48-hour treatment with SymCEP7 elevated *premiR2111d* expression from the already high levels occurring under low-N conditions, and this induction depended on CRA2 (Figure 4E).

### Shoot-applied SymCEP7 promotes nodule number faster and at lower concentrations than root-applied SymCEP7

**Figure 5:**
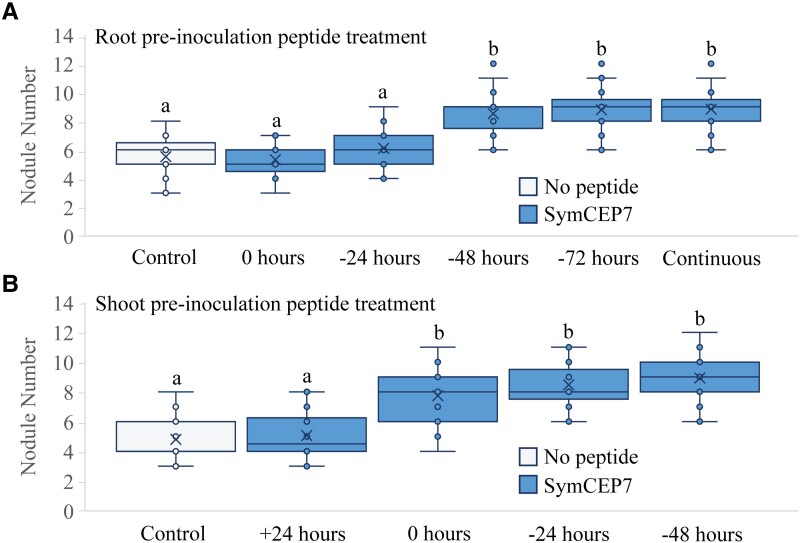
Temporal analysis of the efficacy of root *versus* shoot SymCEP7 application. A, Plants were grown on N-free Fåhraeus medium for four days prior to inoculation. Whole roots were treated with 500 µl of 1 µM SymCEP7 at the time of inoculation, or 24, 48, or 72 h prior to inoculation. Nodules were scored at 14 days post inoculation (dpi). B, Plants were grown on N-free Fåhraeus medium for four days and shoot treated with SymCEP7 (a 10 µl dose at 10 nM) either 24 hours after, at the time of inoculation, or 24 and 48 hours prior to inoculation of Sm1022. Nodules were scored at 14 dpi. In (A) and (B), data are plotted as open circles, centre lines show the median; box limits indicate the 25th and 75th percentiles, crosses represent samples means, whisker boundaries show the 1.5 interquartile range. Significant differences are shown with letters (Two-way ANOVA, *P* < 0.001, *n* = 21–28 plants, one experimental repeat).

A temporal analysis of the effects of shoot- compared to root-applied SymCEP7 was then conducted. The results showed that root-applied SymCEP7 at 10^−6^ M required up to a 48-hour prior exposure to roots to promote nodule number (Figure 5A and B). By contrast, shoot-applied SymCEP7 promoted nodulation at 10^−8^ M without the need for pre-treatment, since it promoted nodulation when applied at the same time as rhizobial inoculation. Given the short duration of the nodulation window, this result implies that (a) shoot-applied SymCEP7 imparts effects to the roots both more rapidly and at lower concentrations than root-applied SymCEP7 and (b) that very few root-applied SymCEP7 molecules are likely to translocate from the roots to the shoots. Consistent with the short duration of the nodulation window (Mohd-Radzman et al., 2016; Figure 1), shoot-applied SymCEP7 did not boost nodule number when applied 24 hours after rhizobial inoculation (Figure 4B).

To explain these temporal and bioactive concentration differences, we tested if the root endodermis barrier slowed the entry of a root-applied CEP7 hormone to the root vasculature, which would consequently cause a slower and diminished translocation to the shoot. Indeed, the application of a FITC-CEP7 derivative to the root required a 48-hour exposure before fluorescence could be detected in the root vasculature (Supplemental Figure 6A–C) and most of the fluorescence remained associated with the outer tissues of the plant root at the point of application. In addition, FITC-CEP7 also accumulated on the outer endodermal surface (arrows in Supplemental Figure 6B), which suggested that the endodermis restricts its entry to the xylem vessels, as would be expected. Collectively, these results support the hypothesis that only a small proportion of root-applied SymCEP7 molecules successfully translocate to the shoot to positively influence nodule number.

### Medicago SymCEP7 also promotes nodule number from the shoot in White Clover and Lotus

**Figure 6:**
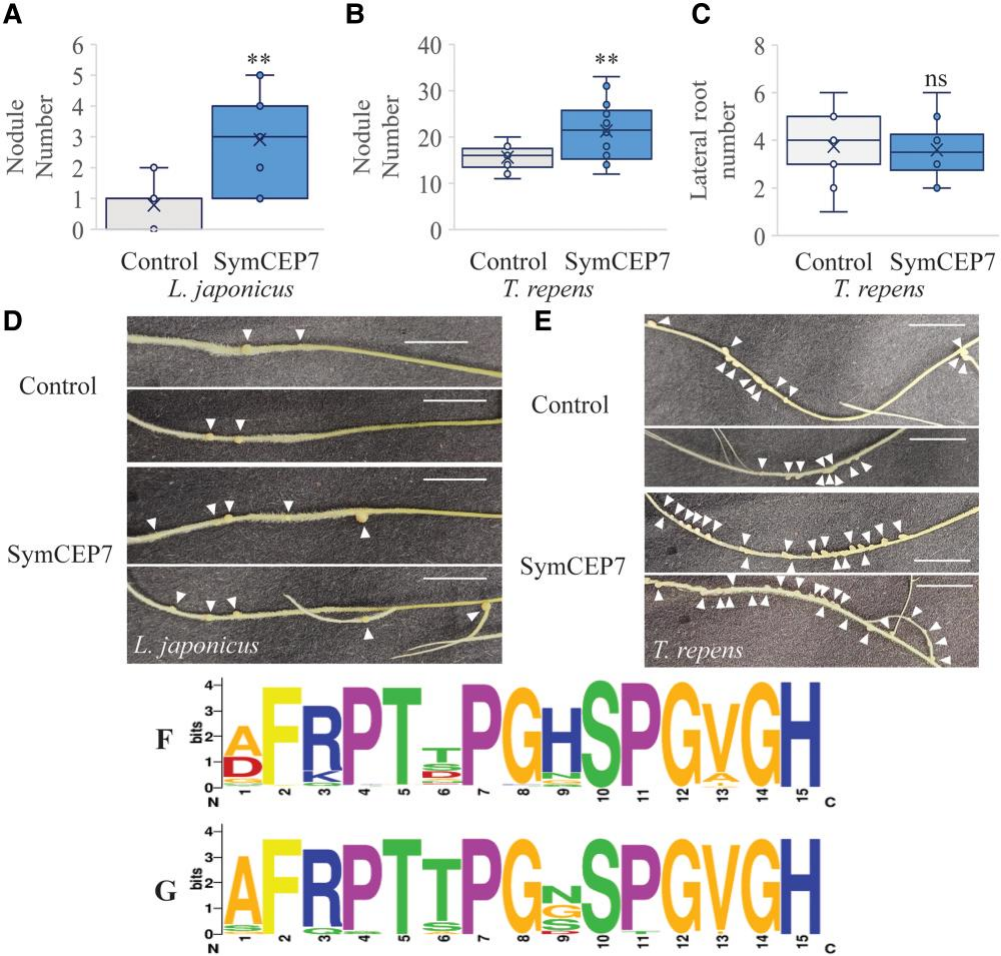
SymCEP7 increases nodule number in a determinate and an indeterminate nodulating legume species. A–D, Lotus and White Clover plants were grown for five days on N-free Fåhraeus medium and the shoots were then treated with 10 µl of water or 10 nM SymCEP7, replenished daily for five days. 24 hours after the first treatment plants were inoculated with *M. loti* or *R. leguminosarum* bv *trifolii*. Root treatment was conducted as described previously. Nodule number in (A) Lotus and (B) White Clover, as well as (C) lateral root number in White Clover were scored three weeks post inoculation. There were so few lateral roots (on average less than 1 per plant) in Lotus that it was not possible to determine if SymCEP7 had a statistical effect on LR number in this species. Data are plotted as open circles, centre lines show the median; box limits indicate the 25th and 75th percentiles, crosses represent samples means, whisker boundaries show the 1.5 interquartile range. Significant differences are shown with asterisks (Student’s t-test, ** *P* < 0.01, *n* = 32 and 36 respectively, one experimental repeat). Representative images of (D) Lotus and (E) White Clover roots harboring nodules (arrows indicate nodules, scale bars = 1 cm). F, The CEP domain motif derived from all legume CEP prepropeptides (*n* = 149 sequences from 10 species) analysed in Ogilvie et al. (2014). G, The Weblogo motif derived from the CEP domain1 sequences from different legumes that cluster in the same clade as CEP7 (*n* = 26 sequences from 9 species). The phylogenetic tree used to identify these CEP domains is shown in the Supplemental Figure S7.

We tested whether the biological action of SymCEP7 was conserved in Lotus or White Clover. Therefore, we applied nM or µM concentrations of the Medicago SymCEP7 to the shoots or roots of Lotus or White Clover, respectively. Shoot- and root-applied SymCEP7 raised nodule number in both legumes but, consistently, a higher concentration was needed for root-applied SymCEP7 to impart biological effects (Figure 6A–E). Like in Medicago, White Clover lateral root number was unaffected by root or shoot application of SymCEP7 (Figure 5C). These results suggest that SymCEP7 orthologues may exist in different legumes.

To identify potential homologs of Medicago CEP7 in other species, a phylogenetic analysis was conducted using the CEP prepropeptide sequences (Supplemental Figure 7A). The prepropeptide sequence of Medicago CEP7 clustered together with Medicago CEP1 and CEP9, as well as with other legume CEP prepropeptides sequences, but not with any CEP sequences from non-legume species (Supplemental Figure 7B), which suggested that the corresponding genes were legume-specific. By contrast, all other Medicago CEP prepropeptide clades had both legume and non-legume homologues.

A Weblogo legume-specific consensus CEP domain 1 motif was derived from the over 30 homologous legume sequences that were retrieved in the Medicago CEP1/CEP7/CEP9 clade. The Medicago CEP1/CEP7/CEP9 clade domain 1 consensus sequence differed from the overall legume CEP consensus motif primarily at the amino acid residue located at position 9. Indeed, the overall legume CEP domain consensus predominantly prefers histidine at position 9, whereas no homolog in domain 1 of the Medicago CEP1/CEP7/CEP9 clade had histidine at this position (Figure 6F and G). The presence of an additional histidine in the acidic environment of the apoplast would impart a net positive charge that would be missing in the domain 1 peptides from Medicago CEP1/CEP7/CEP9 clade. Therefore, the lack of histidine in these legume specific CEPs would impart a differential charge. These results suggest that Medicago *CEP7*, *CEP1* and *CEP9*, and their other related legume sequences in the CEP1/CEP7/CEP9 clade, may produce structurally distinct domain 1 CEP hormones with a symbiosis-associated activity.

## Discussion

Using transcriptional, genetic, biochemical, and microscopy approaches, we show that *CEP7* has a symbiosis-associated expression during the very early stages of nodule initiation, which restricts its expression spatially and temporally to the nodulation zone. The close association of *CEP7* with symbiosis was also confirmed (a) by its elevated expression in *skl*, (b) the requirement of the Sym pathway, NFs, and the CRA2 receptor for SymCEP7 to increase the frequency of infection-associated events and nodule number, (c) by the ability of SymCEP7 to induce *miR2111* expression, (d) by the lack of expression of other CEP genes in *skl*, and (e) the inability of SymCEP7 to reduce lateral root number. It is noteworthy that independent data from Laffont et al. (2020) and Jardinaud et al. (2016) are consistent with these findings. Since NIN co-regulates *CEP7*, as well as the nodule number-suppressing *CLE* genes (Imin et al., 2018; Laffont et al., 2019, 2020), these peptide hormone pathways may compete during the very early stages of infection and nodule establishment to control nodule number positively and negatively, respectively. We propose, therefore, that nodule number is determined in the nodulation zone during the first 48 hours of infection on in vitro grown Medicago by a dynamic interplay between CEP and CLE pathways and that this interplay helps define the size of the nodulation zone and the number of nodules that form. It is likely also that *CEP7* acts in conjunction with the other low-nitrogen induced *CEP* genes, such as *CEP1*, to promote competency to rhizobial infection (Imin et al., 2013; Mohd-Radzman et al., 2016; Laffont et al., 2019; Zhu et al., 2021).

MS analysis indicates that SymCEP7 is the predominant ionized form of the CEP7 hormones produced in vivo. SymCEP7 is the only Medicago CEP hormone identified so far that imparts an exclusive symbiosis-associated phenotype, since all other biologically active group 1 CEP hormones tested to date also inhibit lateral root number (Imin et al., 2013; Mohd-Radzman et al., 2015, 2016; Patel et al., 2018; Zhu et al., 2020). The binding of FITC-labelled SymCEP7 to shoot vascular cells also requires the CRA2 receptor, and competition assays indicate that SymCEP7 can displace a FITC-CEP1 derivative from binding sites in shoot vasculature cells. Collectively, these results reinforce that CRA2 is a receptor partner for CEP peptides, including SymCEP7. The lack of biological activity of the 19 amino acid CEP7 domain 2 peptides may indicate that these species are inactive intermediates that are incompletely processed, in agreement with prior results where three to five amino acid extensions to CEP1 peptides abolished their ability to increase nodule number (Patel et al., 2018).

In agreement with previous studies (Patel et al., 2018; Lee et al., 2021), CEP7 hormones with different structures can have distinct biological activities, with only SymCEP7 holding a symbiosis-associated activity. Remarkably, SymCEP7 and its stereoisomer CEP7 D1: HyP4, 11 have distinct biological activities, as CEP7 D1 HyP4, 11, along with CEP7 D1 HyP4, 7, 11 have the additional ability to reduce lateral root number. This indicates that the location and number of hydroxyl proline residues influences CEP biological activity. In addition, analysis of phylogenetic data based on CEP prepropeptide sequences identified a legume specific CEP1/CEP7/CEP9 clade that preferred a non-charged residue (e.g. Serine, Asparagine) at position 9, as opposed to a strong preference for histidine at the same position in CEPs that had non-legume homologues. Combined with prior structure-function data (Patel et al., 2018), these results suggest that the amino acid at position 9 might also influence CEP biological activity. Indeed, histidine often plays important roles in ligand receptor and protein-protein interactions in animals (Rötzschke et al., 2002) due to the unique pH-dependent charge state of its side chain. Unlike all other amino acids, histidine is positively charged at an acidic pH typical of the apoplast but has no charge as pH rises towards neutral. Therefore, there is likely a net differential charge in CEPs with or without histidine at position 9 in the apoplast. Recently, the interaction between the plant root growth factor (RGF) peptide and its cognate receptor (RGFR) was found also to be pH-dependent (Liu et al., 2022).

Prior grafting and split-root experiments indicated that CEP hormones act systemically on nodule number via the shoot CRA2 receptor, and locally via the root CRA2 on lateral root number (Huault et al., 2014; Mohd-Radzman et al., 2016; Laffont et al., 2019, 2020), but no one has shown that applying CEPs to shoots is an effective way to bypass the commonly-used technique of applying CEPs to roots. The results showed that the direct application of SymCEP7 to shoots at physiological concentrations promotes nodule number at mid pM to mid nM levels, whereas the application of µM levels of SymCEP7 to roots was required to achieve the same effect on nodule number. Therefore, shoot-applied SymCEP7 is bioactive at up to a 10,000-fold lower concentration (i.e. as low as 10 pM) than root-applied SymCEP7, which rapidly loses its biological effect below µM levels. This result is consistent with quantitative estimates of the concentration of CEP hormones in vivo (Mohd-Radzman et al., 2015) as well as with multiple studies showing that CEP hormones promote nodulation from the shoot (Huault et al., 2014; Laffont et al., 2019, 2020). Furthermore, we found that the application of µM levels of SymCEP7 to shoots had the reverse effect of applying pM to nM range concentrations by suppressing nodule number and delaying their emergence. This result is indicative of a typical bell curve dose-response where an excessive hormone concentration leads to opposite effects to those imposed by a physiologically-relevant hormonal concentration (van Noorden et al., 2006). The higher efficiency of shoot-applied SymCEP7 is also reflected by the lack of need to pre-expose this peptide to shoots to enhance nodule number compared to root-applications, which require 48 hours additional pre-exposure to promote nodule number. In addition, shoot-applied SymCEP7 induces the expression of *premiR2111d* in the time period where nodule number is promoted transiently in the nodule initiation window, which is maximally susceptible for a ∼48-hour period after rhizobial inoculation (Mohd-Radzman et al., 2016).

The applications of FITC-labelled CEP7 variants to roots revealed a slow diffusion into the root vasculature combined with an accumulation at the endodermis, which suggested that the well-known root endodermis diffusion barrier likely allows only a very small proportion of root-applied SymCEP7 hormone molecules to enter the root vascular tissue and translocate to the shoot. An alternative explanation could be that SymCEP7 is also subjected to proteolytic degradation prior to entering the root vascular tissue. By contrast, the lack of a Casparian strip in the shoots of Angiosperms (Geldner, 2013) may enable the CEP peptides to gain more ready access to the vascular tissue where the CRA2 receptor is located, which may contribute to the greater effectiveness of shoot- compared to root-applications of SymCEP7 or CEP1. It is noteworthy that the higher speed and sensitivity of SymCEP7 or CEP1 peptide application to shoots may have biotechnological applications in agriculture aiming to promote root nodulation using externally-applied peptide formulations.

Phylogenetic analyses suggest that the prepropeptides encoded by Medicago *CEP7*, *CEP1* and *CEP9* genes are members of a legume specific clade. In contrast, the remaining Medicago CEP prepropeptide family members share homology both with sequences from legumes and non-legumes. These legume-specific clade genes encode distinctive domain 1 CEP hormones with uncharged (Asn, Gly, Ser), or in rare instances, a negatively charged (Asp), residues at position 9, whereas His occurs predominantly at position 9 in CEP sequences outside of this legume-specific clade. The presence of an uncharged amino acid at position 9 of SymCEP7 (Ser), combined with its specific pattern of hydroxyl proline modifications, may form the basis for its ability to have a symbiosis-associated activity. It is possible that members of the *CEP1*/*CEP7*/*CEP9* clade have evolved specific CEP structures to play a more specialized role in controlling root nodulation.

**Figure 7:**
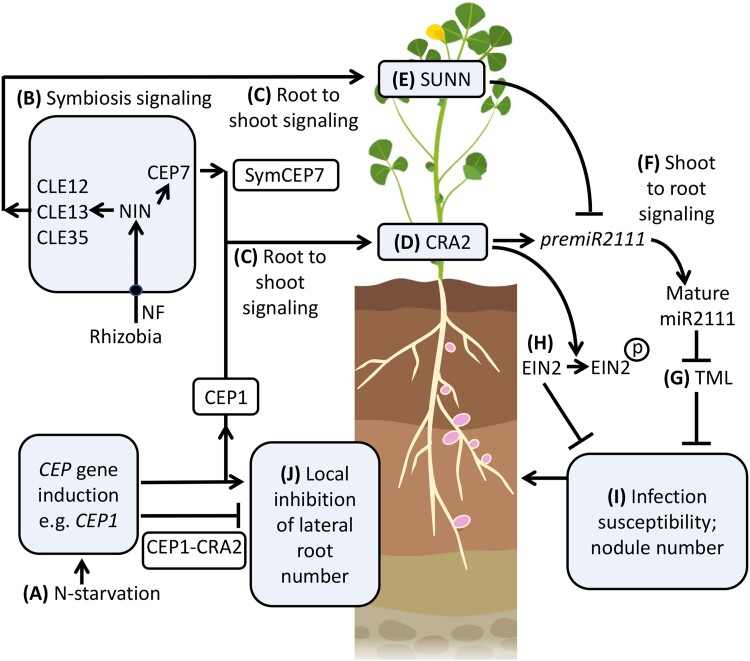
A model for SymCEP7 promotion of nodulation and CEP regulation of lateral root number. A, C, Under N-starvation most Medicago *CEP* genes, (e.g. *CEP1)*, are up-regulated and translocate from the root to the shoot to interact with CRA2. B,C,*CEP7, CLE12, 13 and 35* expression is triggered by rhizobial Nod Factors (NF) and the NIN transcription factor. Subsequently the mature CEP and CLE peptides are secreted to enter the xylem stream to translocate from the root to the shoot to interact with CRA2 or SUNN. D,E, CEP1 and SymCEP7 promote *premiR2111* expression whereas CLE12, 13 and 35 suppress *premiR2111* expression. F,G, Mature miR2111 translocates from the shoot to roots to regulate TML levels. H, Concurrently, CEP1-CRA2 signaling leads to the phosphorylation of EIN2, which reduces ethylene signaling. It is not known if CEP1-CRA2 control of EIN2 phosphorylation status is controlled locally or systemically (I) Reduced TML and ethylene signaling in roots increases infection susceptibility and nodule number. A, J, N-starvation induced CEPs (e.g. CEP1) inhibit lateral root number by a localized interaction with CRA2 in the root. Model based on data from Imin et al. (2013, 2018), Mohd-Radzman et al. (2016), de Bang et al. (2017), Boschiero et al. (2020), Patel et al. (2018), Laffont et al. (2020), Gautrat et al. (2020), Zhu et al. (2020, 2021), Moreau et al. (2021), Mens et al. (2021) and this study. Created with BioRender.com. Black circle = NF receptor.

The current model of the systemic regulation of nodulation is that CEP hormones produced under low N (e.g. derived from *CEP1*) prime the plant for susceptibility to compatible rhizobia by increasing the levels of mature *miR2111* which downregulates *TML* transcript levels in the root (Gautrat et al., 2020; Moreau et al., 2021; Okuma and Kawaguchi, 2021), whereas triarabinosylated CLE hormones counteract this pathway by interacting with the SUNN receptor to inhibit *miR2111* transcription (Gautrat et al., 2020, 2021). Our study updates this model (Figure 7), based on the finding that the *CEP7* nodulation-promoting gene is transiently expressed in symbiotic tissues during the nodule initiation window, which would allow the SymCEP7 hormone to translocate to the shoot to counteract the negative autoregulation of nodulation pathways that are also triggered by rhizobial inoculation (Imin et al., 2018; Ferguson et al., 2019; Laffont et al., 2020). Finally, we highlight that the CEP-dependent inhibition of lateral root number is root-localized and independent of SymCEP7. Overall, we propose that SymCEP7-like hormones may have evolved in legumes to increase the duration of susceptibility to nodulation without compromising lateral root number and growth. This process would enable legumes to maintain a capacity to acquire nutrients other than N via lateral root foraging activity under symbiotic conditions.

## Materials and methods

### Biological materials and growth conditions

The wild type Medicago (*Medicago truncatula*), Jemalong A17, was used in this study, along with the mutants *cra2-11* (Laffont et al., 2019), *dmi1, dmi2* (Ané et al., 2004), *nin1-1* (Marsh et al., 2007), *nsp1*, *nsp2*, (Smit et al., 2005), *sickle1-1* (Penmetsa and Cook, 1997), and *nfp* (Amor et al., 2003). Medicago seeds were germinated and vernalized as described previously (Mohd-Radzman et al., 2016). Following germination, seedlings were transferred to growth plates containing 50 ml of Fåhraeus medium (modified from Vincent et al., 1970) in 150 mm plates poured at an angle of 15°. Six to seven germinated plants were positioned near the top edge of the set agar (Gelita Australia Pty Ltd) so that, once grown, the shoots were not in contact with the agar medium. Plates were then sealed with parafilm, with a 70 mm slit cut at the top of the plate to enable air exchange. Plants were vertically positioned, with the roots in the lower half of the plates covered by black cardboard to maintain a darker environment. Plants were maintained at 23°C, with a photon flux density of 100 µmol m/s and a 16-hour photoperiod.

Prior to inoculation with *Sinorhizobium meliloti*, all plants used for nodulation assays were starved of N for three days, unless otherwise specified. *S. meliloti* cultures were grown overnight at 28°C with continuous shaking in BMM medium (Vincent, 1970). After centrifugation and adjustment of the density (OD_600_ = 0.01), roots were then inoculated with 500 µl of culture. Bacterial strains used for nodulation experiments were *S. meliloti* strain 1022 (Terpolilli et al., 2008), *Sinorhizobium medicae* ABS7M (Larrainzar et al., 2015), or the non-nodulating *S. meliloti* strain SL44 (Mitra et al., 2004), which is unable to make NFs due to a *nodDABC* deletion. Bacterial strains used for cloning and transformation into plants were DH5α (ThermoFisher) and ArQUA1 (Boisson-Dernier et al., 2001), respectively.

For cross species CEP activity analyses, the indeterminate legume White Clover (*Trifolium repens*) cv. Haifa and the determinate legume Lotus (*Lotus japonicus*) Gifu B-129 were used. White Clover and Lotus were germinated, grown, and inoculated with either *Rhizobium leguminosarum* bv. *trifolii* ANU843 (Djordjevic et al., 1985) or *Mesorhizobium loti* MAFF303099, respectively, as described in Gauthier-Coles et al. (2019).

### CEP hormone and NF treatments

The synthetic peptide hormones used were CEP1D1: HyP4, 11 (AFQhyPTTPGNShyPGVGH), CEP7 D1: HyP4, 11 (SFRhyPTTPGSShyPGVGH), CEP7 D1: HyP7, 11 (SymCEP7; SFRPTThyPGSShyPGVGH), CEP7 D1: HyP4, 7, 11 (SFRhyPTThyPGSShyPGVGH), CEP7 D2 HyP4, 7, 11 *N*-ext (RSDDGFKhyPTNhyPSHShyPGVGH), and CEP7 D2 HyP11 *N*-ext (RSDDGFKPTNPSHShyPGVGH). CEP1D1:HyP4, 11 and SymCEP7 were also synthesized with an *N*-terminal fluorescein isothiocyanate (FITC) group to visualize binding to vascular tissue (Lee et al., 2021). All peptides used in this study were synthesized by GL BioChem Ltd., Shanghai, China at >95% purity and their structure validated quantitatively and qualitatively by HPLC (CXTH MC3000, Beijing) and MS (SHIMADZU LCMS02010EV and SHIMADZU LCMS02020, Japan) prior to use. All peptides were diluted in sterile water and filter sterilized prior to use. Sterile Milli Q water was used for standard dilutions (Millipore, Bedford, MA).

For peptide activity assays, synthetic peptides were either added to the growth medium immediately prior to pouring plates or pipetted as 500 µl aliquots onto roots as specified in the figure legends. Peptides were applied to the petioles of cotyledons in 10 µl droplets (Figure 4A). Shoot application was maintained over five days by replenishing the 10 µl droplet daily. The surface tension of the peptide containing solution fixed it in place for the duration of the experiment without spillage.

Synthetic *S. meliloti* and *Bradyrhizobium japonicum* NFs were prepared as a master stock of 1 mM in DMSO. NFs were diluted to 10 nM with sterile water and pipetted onto whole roots (200 µl) grown on N-free Fåhraeus medium for 3 days. Methylene blue staining was used to visualize infection threads as previously described (Mohd-Radzman et al., 2016).

### Gene expression analysis by Rt-qPCR

Up to 100 mg of tissue was snap frozen in two mL tubes (Sarstedt, Cat#NC0418367 Fisher Scientific) with twenty 2 mm Bashing Beads (Zymo). Frozen samples were ground at −80°C using an in house automated cold grinding robot “Frosty” (Adam Carroll, RSB, Australia) using a custom protocol. Samples were ground for 2 min, alternating 30 s at maximum speed and 30 s at rest to limit temperature increases due to friction. Total RNAs were isolated from frozen roots with the RNeasy Plant mini kit (Qiagen, http://www.qiagen.com/) according to manufacturer's instructions. Small RNAs were isolated using the protocol described in Li et al. (2014) and prepared for cDNA synthesis as described in Gautrat et al. (2020). Total RNAs (1.5 µg) were used for cDNA synthesis with SuperScript™ IV VILO™ Master Mix with ezDNase™ Enzyme (Invitrogen). RT-qPCR experiments were performed on a LightCycler 480 apparatus using the LightCycler480 SYBR Green I Master Kit (Roche Diagnostics, http://lifescience.roche.com) according to manufacturer's instructions. Cycling conditions were as follows: 95°C for 5 min, and then 40 cycles at 95°C for 15 s, 60°C for 15 s, and 72°C for 15 s. A dissociation curve (55–95°C) was performed to assess the specificity of the amplification. The expression of genes of interest was normalized against the reference gene MtUBI previously selected using the Genorm software (https://genorm.cmgg.be/). Primers used for RT-qPCR for the *ubi* control gene: forward primer (5′-3′) (GAACTTGCATGGGTCTTGA), reverse primer (5′-3′) (CATTAAGTTTGACAAAGAGAAAGAGACAGA); *CEP7* forward primer (5′-3′) (CCGGATGTTGAGGTTTTTGT) and reverse primer (5′-3′) (GGCCAACTCCAGGACTATGA) and *premiR2111d* forward primer (5′-3′) (TCTGCATCCTGAGGTTTATAGCA) and reverse primer (5′-3′) (GGCATTAAGGAAAGGATAATCTGCA). Cycle threshold (Ct) values obtained for the reference gene were averaged prior to calculate the ratios of genes of interest onto reference genes. These ratios were calibrated relative to the experimental control condition (WT genotype and/or untreated mock control).

### In planta isolation, extraction, and identification of CEP7 peptides from hairy root cultures

High-density axenic hairy root cultures harboring an empty vector were prepared for MS analysis. Supernatants were extracted using *o*-chlorophenol-acetone precipitation followed by size exclusion chromatography with concentration/desalting by reversed-phase chromatography (Mohd-Radzman et al., 2015; Patel et al., 2018). After lyophilization, samples were resuspended in 400 µl of 3% acetonitrile with 0.1% formic acid (v/v) prior to nano-liquid chromatography-electrospray ionization (LC-ESI)-MS/MS analysis, and an aliquot was used for MS analysis.

### Nano-LC-ESI-MS/MS

CEP7 hormones were identified using an unbiased MS procedure, fully described in Patel et al. (2018). Full MS scans were acquired in the Orbitrap mass analyzer over the range m/z 350–1800 with a mass resolution of 70,000 (at m/z 200). The 10 most intense peaks with a charge state ≥1 were fragmented in the high energy C-trap dissociation collision (HCD) cell to generate tandem mass spectra with a mass resolution of either 17,500 or 35,000 at a m/z of 200 The raw files generated were analyzed on Proteome Discoverer 2.1 using Sequest HT as a search engine as described previously. The fragmentation type used was a high energy collision dissociation (HCD), and the precursor and fragment mass tolerance were set at 10 ppm and 0.08 Da, respectively. CEP7 sequences were identified using the unbiased bioinformatic strategy from Patel et al. (2018).

### Visualization of FITC-SymCEP7 binding to semi-purified vascular tissues

The binding of FITC-CEP7 and FITC-CEP1 to semi-purified shoot vascular tissues was performed according to the method of Lee et al. (2021) and observed using confocal microscopy (Zeiss Confocal LSM 780) with the argon laser excitation set to 485 nm to image the FITC-labelled peptide binding to vascular tissue. The fluorescence spectra of FITC and vascular tissue autofluorescence were determined using the spectral scanning module where the FITC emission peak was at 525 nm and vascular tissue autofluorescence was ∼600 nm (Lee et al. 2021). All images were representative from three independent experiments. Competition experiments using the SymCEP7 and FITC-CEP1 hormones were performed by mixing equimolar amounts of each peptide species (Lee et al., 2021).

### Visualization of FITC-CEP7 after application to roots

To visualize the ability of CEP7 to enter roots, freshly germinated wild type seedlings were transferred to Fåhraeus medium containing 5 mM KNO_3_ supplemented with 1% (w/v) sucrose. The plates were wrapped in foil immediately to maintain a dark environment to minimize the basal autofluorescence in plant roots and grown at 22–25°C. After four days, 500-µl of 1 µM FITC-CEP7 Hyp4, 11 was pipetted onto roots, minimizing exposure to light throughout. Roots embedded in agar were sectioned using a vibratome (VT1200S, Leica) at 24-hour intervals for visualization using confocal microscopy (Zeiss Confocal LSM 780) with the argon laser excitation set to 485 nm and the FITC emission peak was captured at 520–525 nm essentially as described in Lee et al. (2021). FITC emissions were distinguished from background autofluorescence using the spectral scanning module. For imaging Master gain was set to 100 for root sections and increased to 450 for 10 × magnifications of the vascular tissue.

### Phylogenetic analysis

Comparative sequence logos (Schneider and Stephens, 1990) were generated using the WebLogo tool (Crooks et al., 2004) by identifying and aligning all CEP domains from the plants of Fabaceae lineage and compared with CEP domains identified as homologues of MtCEP7 in Ogilvie et al. (2014). The phylogenetic tree supporting the WebLogo alignment appears in Ogilvie et al. (2014).

### Statistical analyses

For RT-qPCR, each biological replicate contains pools of plants from a minimum of three plates (approximately seven plants per plate), and three technical repeats were performed. For phenotyping assays, biological replicates are either individual roots or shoots where specified. Student’s t-tests and two-Way ANOVAs were applied on normally distributed data, with Tukey-Kramer's post-hoc tests unless stated otherwise. Regional root expression was analyzed using linear mixed-effects modeling (Bolker et al., 2009), where time points and tissue types (root zones) were entered into the model as fixed effects, while biological and technical replicates were entered as random effects (see Supplemental Method 1). *P* values were generated from the association between random effects (changes in expression) as a function of individual fixed effects (time or tissue) and their interaction (time × tissue). Statistical analyses were performed in Microsoft Excel (2016) or R Studio (RStudioTeam, 2015) using the lme4 package (Bates et al., 2014).

### Accession numbers

Sequence data from this article can be found in the GenBank/EMBL data libraries under accession numbers MT35v5_AC233112_1013 (CEP7), MT35v5_contig_59554_1 (CEP1), MtrunA17Chr7g0240286 (miR2111), Medtr3g110840.1 (CRA2).

## Supplementary Material

kiad012_Supplementary_Data

## Supplemental data

The following materials are available in the online version of this article.

**Supplemental Figure S1**. *CEP7* expression is up-regulated during the early stages of infection and is hyper-expressed in *skl*.

**Supplemental Figure S2**. Analysis of *CEP7* expression and effects of SymCEP7 treatment.

**Supplemental Figure S3**. Other CEP7 hormone structures identified in the hairy root culture exudates.

**Supplemental Figure S4**. SymCEP7 increases root hair infection events.

**Supplemental Figure S5**. The effects of CEP structure on biological activity.

**Supplemental Figure S6**. Root application of FITC-CEP7 requires a 48 hour exposure to be detected in the root vasculature.

**Supplemental Figure S7**. Phylogenetic analysis of the CEP family.

**Supplemental Method S1**. Linear mixed effects modeling.
