# Supplementary material for: The Medicago SymCEP7 hormone increases nodule number via shoots without compromising lateral root number
Source: Plant Physiol. 2023 Jan 19;191(3):2012–26. doi: 10.1093/plphys/kiad012 (PMC10022606; doi:10.1093/plphys/kiad012)
Supplement: kiad012_Supplementary_Data [file kiad012_supplementary_data.zip › PP2022RA01172DR1_Supplemental_Figures.pdf]

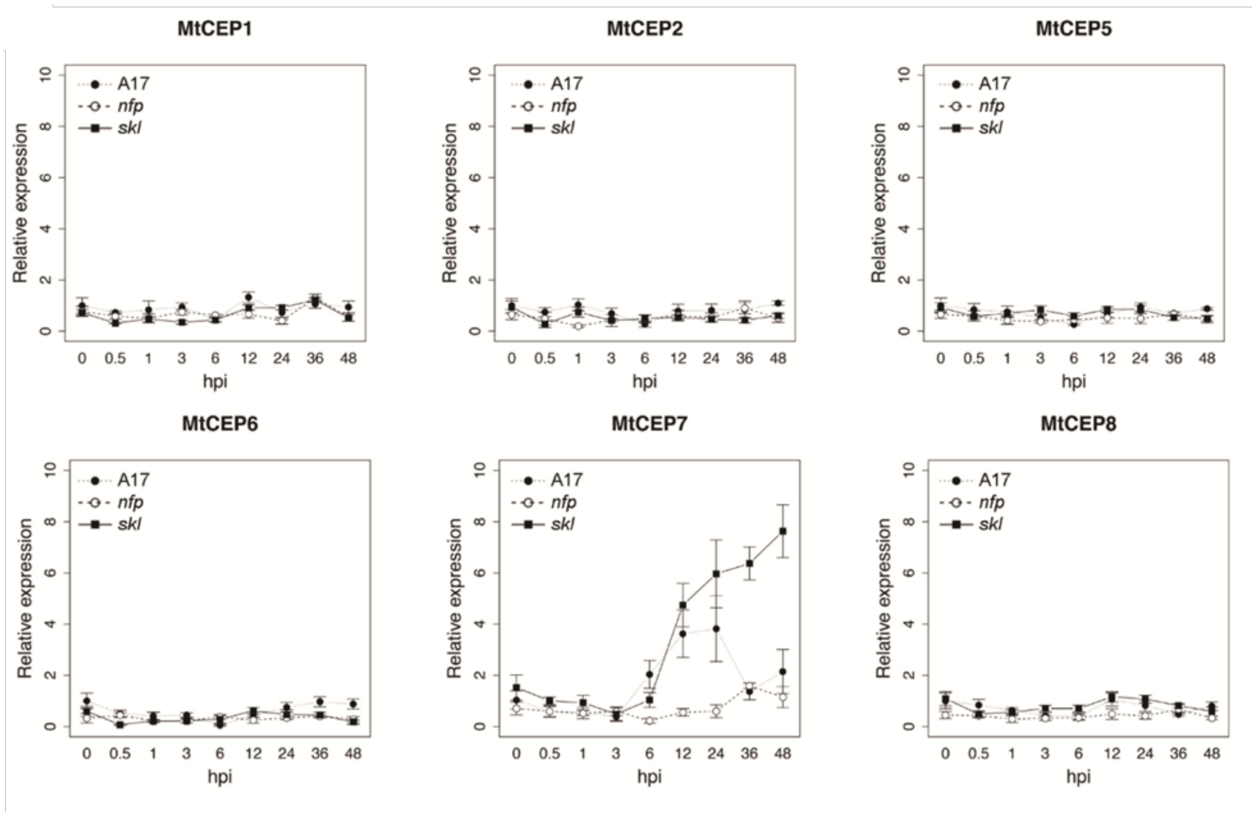

Supplemental Figure S1. ***CEP7*** **expression is up-regulated during the early stages of infection and is hyper-expressed in** ***skl*****.** RNA-seq data showing Medicago *CEP1*, *CEP2*, *CEP5*, *CEP6*, *CEP7*, and *CEP8* expression in wild type (A17), *nfp* and *skl* roots infected with *S. medicae* strain ABS7M at nine time points over 48 hours (Larrainzar et al, 2015).

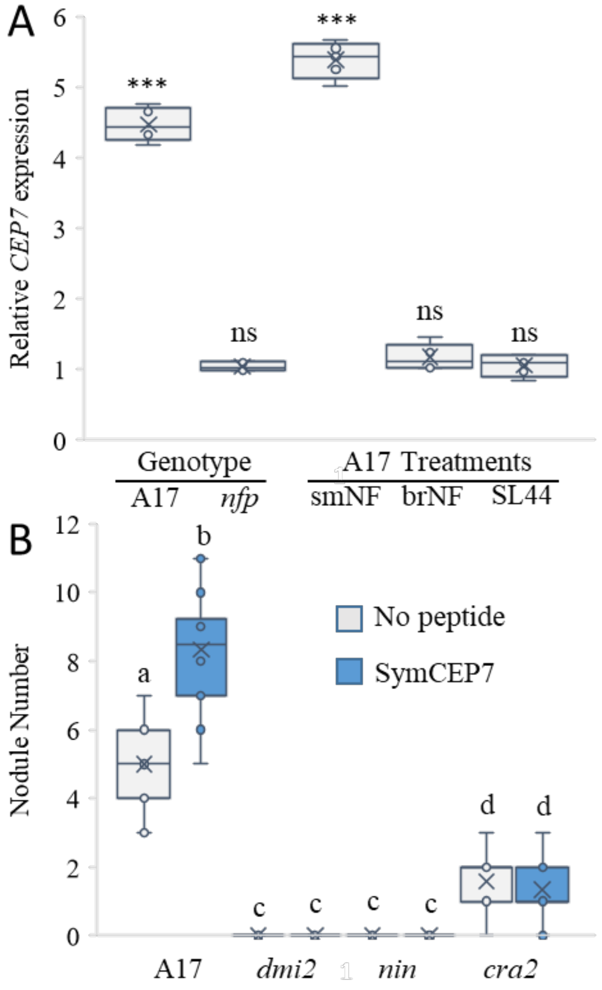

Supplemental Figure S2. Analysis of *CEP7* expression and effects of SymCEP7 treatment. (A) Genotype effects: *CEP7* expression in wild type (A17) or *nfp* after inoculation with *S. meliloti* 1022. Treatment effects: Expression of *CEP7* in A17 24 hours after treatment with sinorhizobial NF (smNF), bradyrhizobial NF (brNF), or the non-nodulating strain *S. meliloti* strain, SL44 (deleted for *nodDABC*). Significant differences are shown with asterisks (*** P<0.001, Student's *t*-test, n=4 biological replicates containing 18 roots each, two experimental repeats). **(B)** Nodule number in Medicago strains after SymCEP7 treatment to roots. A17, *dmi2*, *nin*, and *cra2* seedlings were grown on N-free Fåhraeus medium supplemented with or without SymCEP7 for three days and then inoculated with Sm1022. Nodules were scored 14 days post inoculation. Significant differences are shown with letters (Two-way ANOVA, p<0.001, n=18-24 roots). Data were plotted as open circles, center lines show the median; box limits indicate the 25th and 75th percentiles, crosses

represent samples means, whisker boundaries show the 1.5 interquartile range.

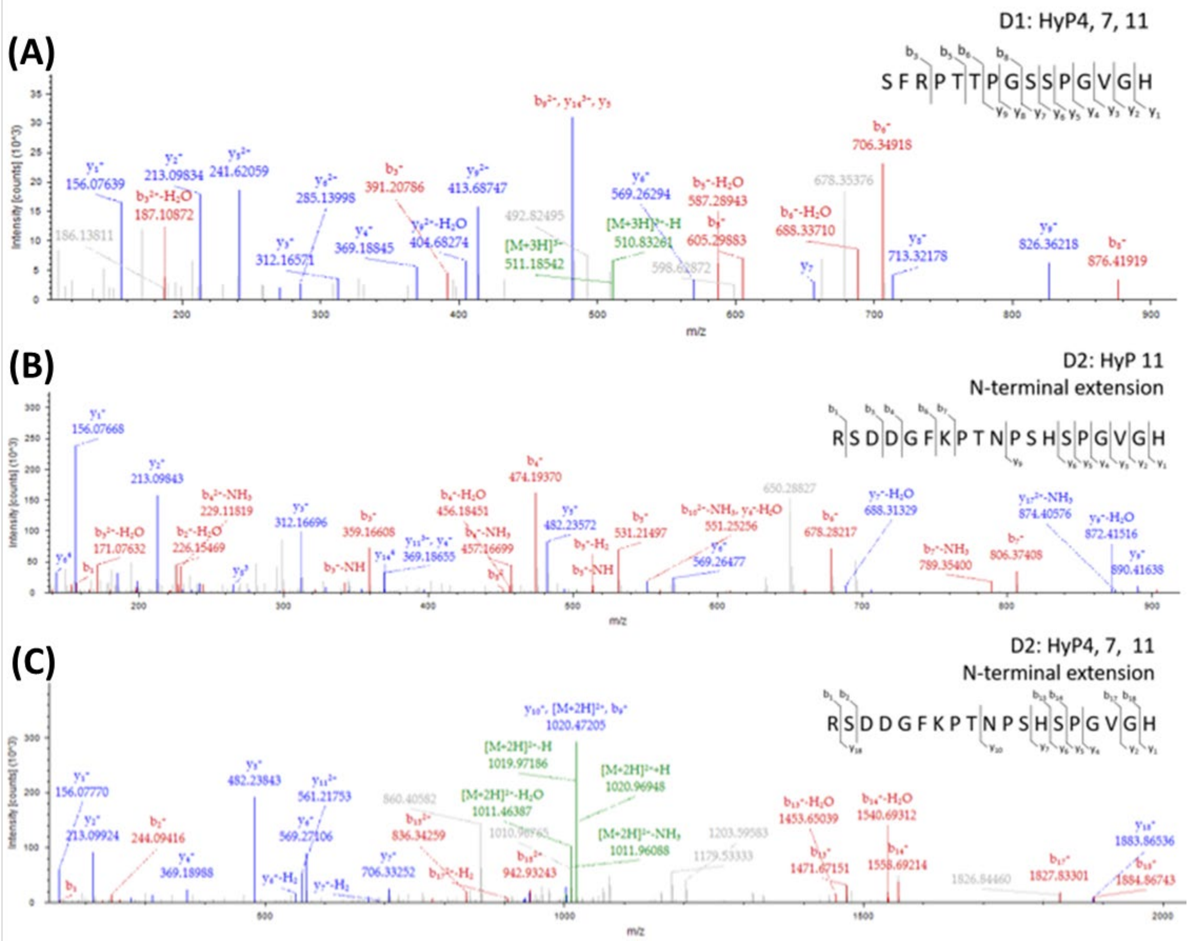

Supplemental Figure S3. Other CEP7 hormone structures identified in the hairy root culture exudates. (A-C) MS-MS spectrum of additional CEP7 species identified in hairy root culture exudates by high accuracy MS (Patel et al 2018). Highlighted in blue and red, respectively, are the matched y and b ions that correspond to the CEP7 domain 1 or domain 2 structures found *in vivo*. CEP7 D1:Hyp4,7,11 **(A)**, CEP7 D2:HyP11 **(B)**, and CEP7 D2:HyP4,7,11 **(C)**. Species in **(B)** and **(C)** have a four amino acid N-terminal extension.

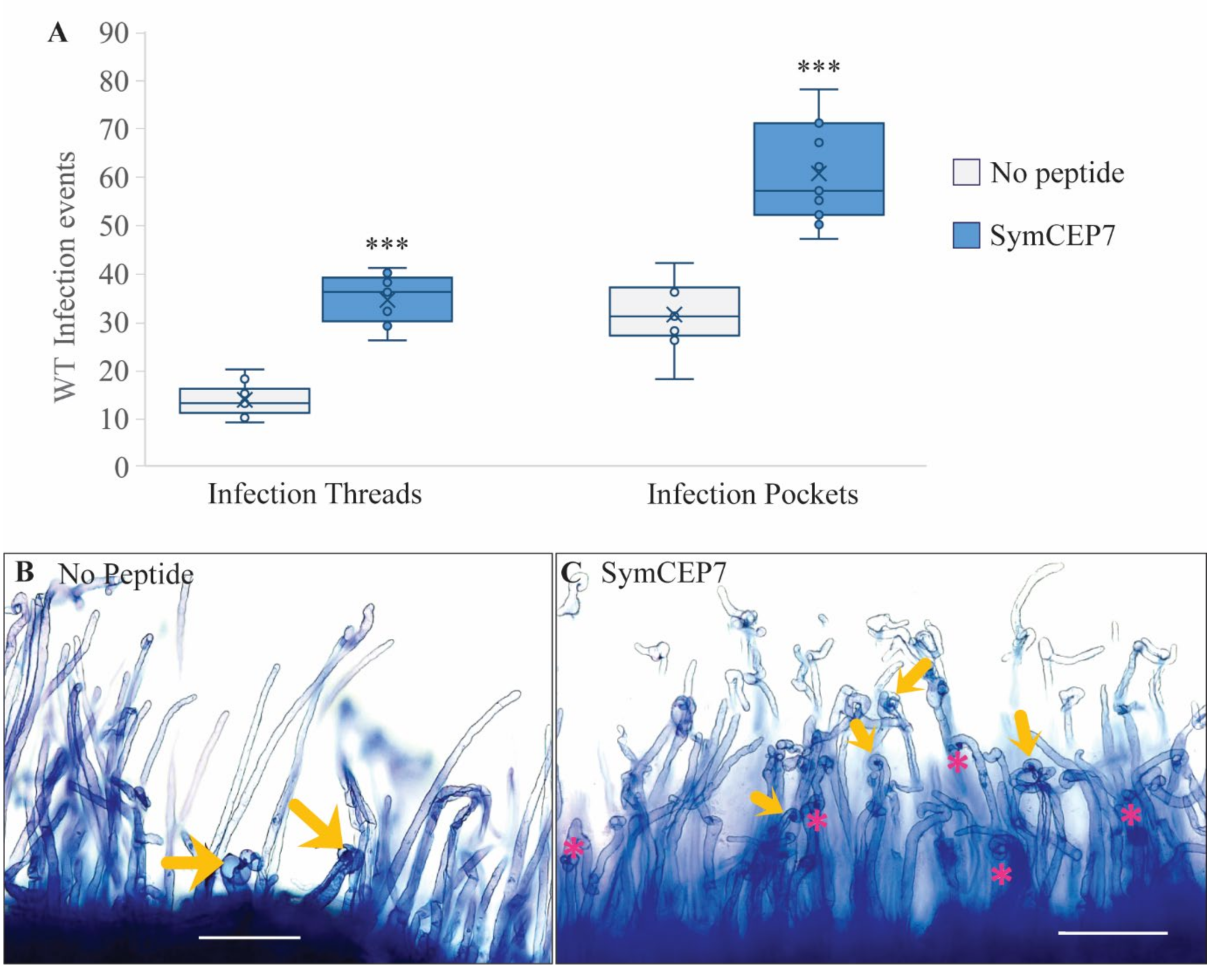

Supplemental Figure S4. SymCEP7 increases root hair infection events. (A-C) Plants were grown on N free Fåhraeus medium supplemented with or without 1 µM SymCEP7. Infection pockets and threads were scored for the whole root 4 days post inoculation with Sm1022 (dpi). **(A)** Effect of SymCEP7 on the number of infection threads and infection pockets. Significant differences shown by asterisks (*** $P<0.001$, Student's t-test, n=24 roots, two experimental repeats). Data are plotted as open circles; center lines show the median; box limits indicate the 25th and 75th percentiles; crosses represent sample means, whisker boundaries show the 1.5 interquartile range. **(B, C)** Representative images showing frequency of infection pockets (pink asterisks) and infection threads (yellow arrows) in **(B)** untreated and **(C)** SymCEP7-treated plants, 4 dpi, scale bars = 100 µm.

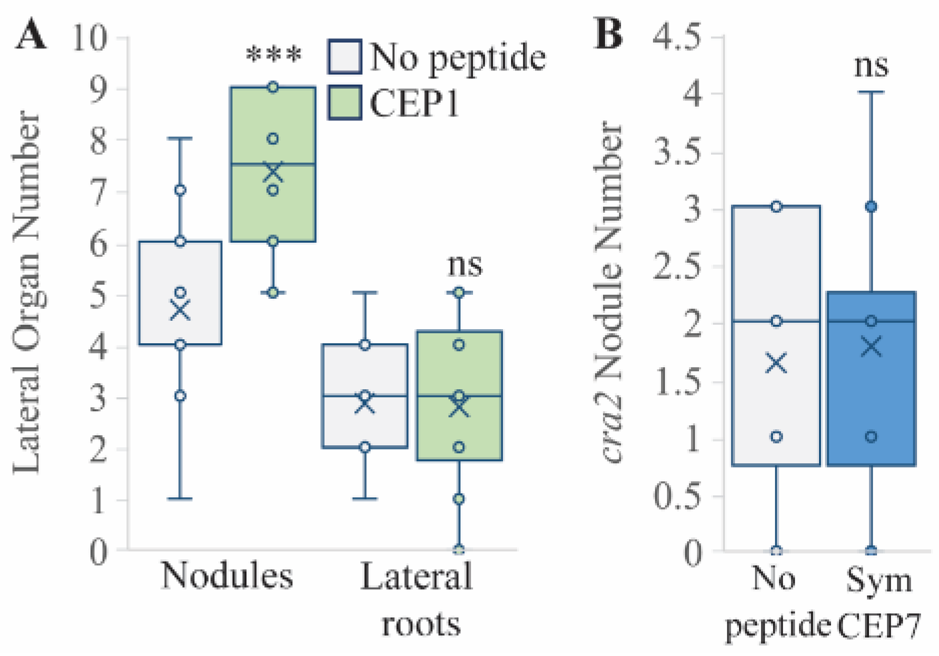

Supplemental Figure S5. The effects of CEP structure on biological activity. (A) Effect of shoot-applied CEP1 at 10^{-8}M on nodule and lateral root number in wild-type plants (Student's t-test, *** $p<0.001$; n = 18-24 plants). **(B)** Effect on nodule number after SymCEP7 treatment of *cra2* shoots (Student's t-test, $p=0.756$, n=18 plants, two experimental repeats). For **A** and **B** data are plotted as open circles; center lines show the median; box limits indicate the 25th and 75th percentiles; crosses represent samples means, whisker boundaries show the 1.5 interquartile range.

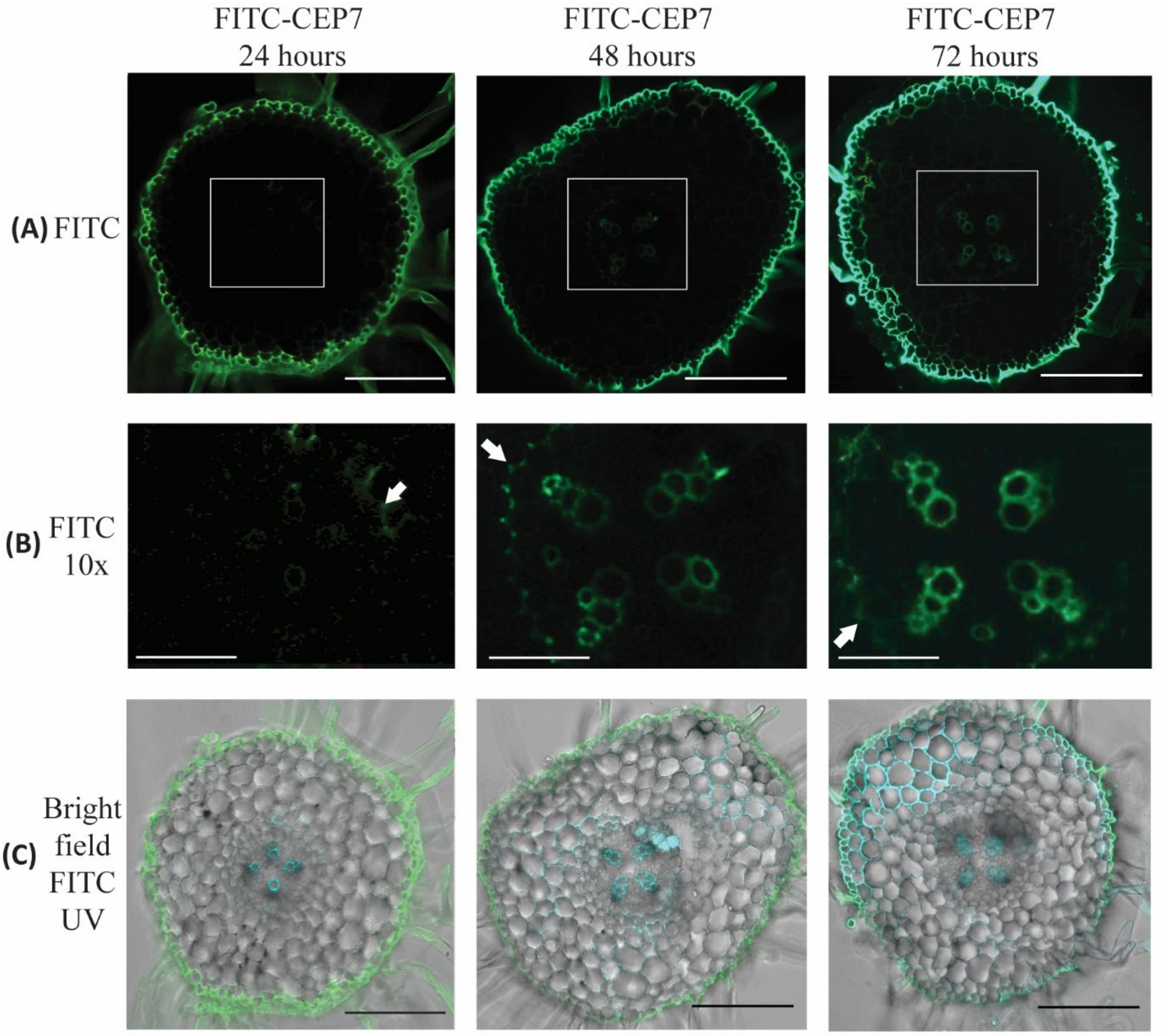

Supplemental Figure S6. Root application of FITC-CEP7 requires a 48 hours exposure to be detected in the root vasculature. (A-C) Plants were grown on N-free Fåhraeus medium supplemented with 1% sucrose in the dark for five days, then treated with 500 µl of at 10^{-6}M FITC-CEP7 Hyp4, 11 for 24, 48 and 72 hours. Roots were sectioned and imaged using confocal microscopy **(A, B)** or via bright field (emission measured at 520-525 nm with Master gain set at 100 for panels in **A** and at 450 in panels in **B**) **(C)**. **B** is a 10x magnification of the white boxes in **A** with a higher gain. White arrows indicate accumulation of FITC signal at the outer face of the endodermis. Scale bars = 150 µm in **A** and **C** and 15 µm in **B**.

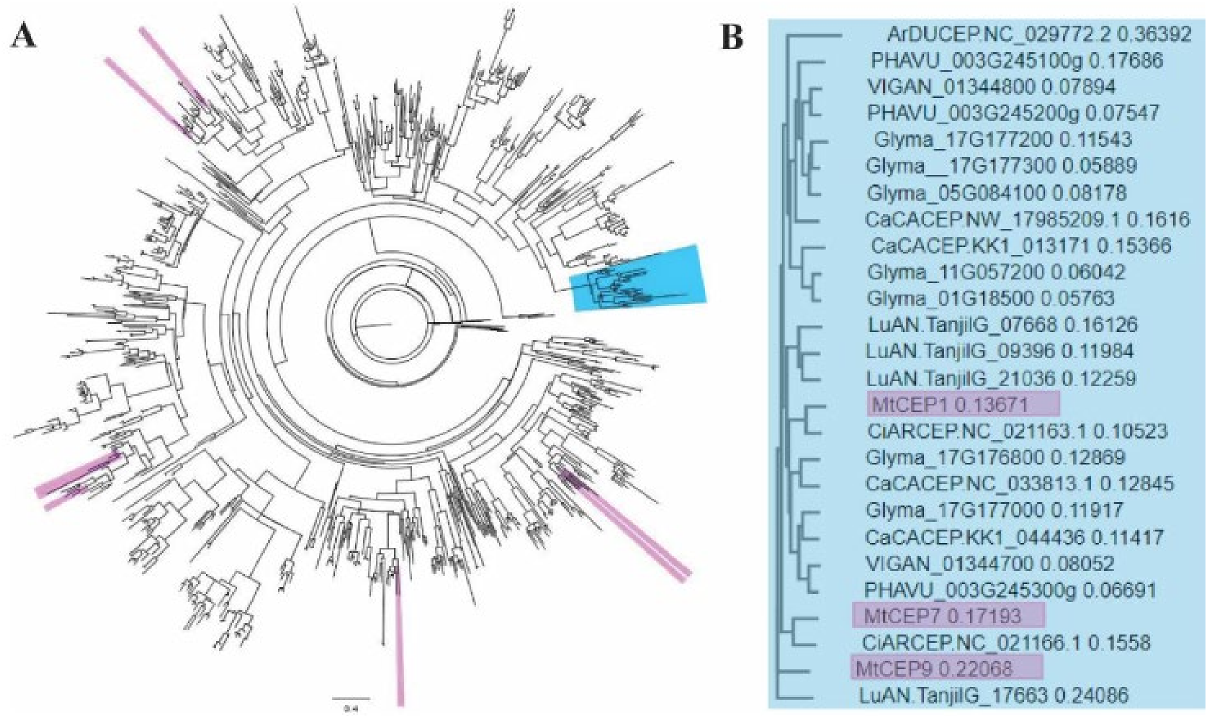

Supplemental Figure S7. Phylogenetic analysis of the CEP family. (A) Maximum likelihood tree of the full CEP prepropeptide sequences listed in Ogilvie et al. (2014) including 106 species of which ten are legumes. Medicago CEP1 CEP7 and CEP9 prepropeptide sequences belong to the legume-specific cluster highlighted in blue, which was identified in both amino acid and nucleotide sequence-based phylogenies by Ogilvie et al. (2014). All other class 1 Medicago CEP prepropeptides highlighted by the pink clusters grouped both with legume and non-legume CEP sequences. Scale: 0.4 substitutions per site. **(B)** Details of the blue box in **(A)** are shown, with the position of Medicago CEP1, CEP7 and CEP9 highlighted in pink. The legumes represented in this cluster include: *Arachis duranensis* (ArDU), *Cajanus cajan* (CaCA), *Cicer arietinum* (CiAR), *Glycine max* (Glyma), *Lupinus angustifolius* (LuAN), *Phaseolus vulgaris* (PHAVU), and *Vigna angularis* (VIGAN).
