# Supplementary material for: The Medicago SymCEP7 hormone increases nodule number via shoots without compromising lateral root number
Source: Plant Physiol. 2023 Jan 19;191(3):2012–26. doi: 10.1093/plphys/kiad012 (PMC10022606; doi:10.1093/plphys/kiad012)
Supplement: kiad012_Supplementary_Data [file kiad012_supplementary_data.zip › supplemental method 1.pdf]

Supplemental Method 1. Linear Mixed Effects Modeling

Linear Mixed Effects Modelling with R Studio

```
Linear mixed-effects model fit by REML
 Data: pcr24.48
       AIC      BIC    logLik
  45.54848 55.80863 -15.77424

Random effects:
 Formula: ~1 | biorep
        (Intercept)
StdDev: 9.129714e-06

 Formula: ~1 | techrep %in% biorep
       (Intercept)  Residual
StdDev:    0.119777 0.2755481

Fixed effects: y ~ time * tissue
                  Value  Std.Error DF   t-value p-value
(Intercept)    7.267222 0.20922619 24  34.73381  0.0000
time          -0.125176 0.01082455 24 -11.56407  0.0000
tissuert       0.550556 0.29045323 24   1.89551  0.0701
time:tissuert  0.118343 0.01530823 24   7.73065  0.0000
 Correlation:
              (Intr) time   tissrt
time          -0.931
tissuert      -0.694  0.671
time:tissuert  0.658 -0.707 -0.949

Standardized Within-Group Residuals:
       Min         Q1        Med         Q3        Max
-1.3536155 -0.7411676 -0.2525164  0.6869728  1.6589657

Number of Observations: 36
Number of Groups:
             biorep techrep %in% biorep
                  3                   9


Linear mixed-effects model fit by REML
 Data: pcr48.96.120
       AIC      BIC    logLik
  284.9825 305.8399 -133.4912

Random effects:
 Formula: ~1 | biorep
        (Intercept)
StdDev: 2.979494e-05

 Formula: ~1 | techrep %in% biorep
        (Intercept) Residual
StdDev: 3.784605e-06 1.097629

Fixed effects: y ~ time * tissue
                  Value Std.Error DF   t-value p-value
(Intercept)    0.386857 0.6559587 67  0.589758  0.5573
time           0.050503 0.0070570 67  7.156492  0.0000
tissuenz       6.029254 0.9276657 67  6.499382  0.0000
tissuert       6.918548 0.9276657 67  7.458018  0.0000
time:tissuenz -0.035457 0.0099801 67 -3.552773  0.0007
time:tissuert -0.047584 0.0099801 67 -4.767857  0.0000
```

```
 Correlation:
              (Intr) time   tissnz tissrt tm:tssn
time          -0.947
tissuenz      -0.707  0.669
tissuert      -0.707  0.669  0.500
time:tissuenz  0.669 -0.707 -0.947 -0.473
time:tissuert  0.669 -0.707 -0.473 -0.947  0.500

Standardized Within-Group Residuals:
        Min          Q1         Med          Q3         Max
-2.65770582 -0.28019272  0.01759203  0.46394379  1.82187815

Number of Observations: 81
Number of Groups:
             biorep techrep %in% biorep
                  3                   9
```
